# Outcomes of Transurethral Resection of the Prostate (TURP) in High-Risk Cardiac Patients Under Spinal Anesthesia

**DOI:** 10.7759/cureus.90137

**Published:** 2025-08-15

**Authors:** Zabish Mehmood, Muhammad Asad Abdullah, Muhammad Sanan, Hamna Fayyaz, Usama Ahmad, Alishba Iftikhar, Farhan Aslam, Saiher Pervaiz Khanday

**Affiliations:** 1 Urology, Sir Ganga Ram Teaching Hospital, Lahore, PAK; 2 Urology, Bahawal Victoria Hospital, Bahawalpur, PAK; 3 Surgery, Central Park Medical College, Lahore, PAK; 4 Orthopedics, Sir Ganga Ram Teaching Hospital, Lahore, PAK

**Keywords:** cardiac, high risk, patients, spinal anesthesia, transurethral resection of prostate (turp)

## Abstract

Background

Transurethral resection of the prostate (TURP) is the standard surgical treatment for benign prostatic hyperplasia (BPH). However, its safety in high-risk cardiac patients remains a clinical concern, particularly regarding perioperative cardiac complications.

Objective

To evaluate the intraoperative and postoperative outcomes of TURP under spinal anesthesia in high-risk cardiac patients.

Methods

This descriptive observational study included 225 male patients aged ≥50 years with documented high-risk cardiac conditions (American Society of Anesthesiologists (ASA) Class III-IV). All underwent TURP under spinal anesthesia at the Department of Urology, Fatima Jinnah Medical University (FJMU)/Sir Ganga Ram Teaching Hospital, Lahore, from February 2023 to August 2023. Data collected included demographic variables, cardiac comorbidities, intraoperative hemodynamic changes, postoperative cardiac events, incidence of TUR syndrome, reoperation rates, and 30-day mortality.

Results

The mean age of patients was 68.4±7.8 years. Among the 225 patients, the most common cardiac comorbidity was ischemic heart disease, seen in 142 (63.1%) patients, followed by congestive heart failure in 51 (22.7%). Intraoperative hypotension occurred in 63 (28.0%) patients, while bradycardia was observed in 27 (12.0%). Vasopressor support was required in 52 (23.1%) patients. Postoperatively, new-onset arrhythmias developed in 11 (4.9%) cases, acute heart failure in seven (3.1%) cases, non-fatal myocardial infarction in three (1.3%) cases, and TUR syndrome in five (2.2%) cases. Reoperation was necessary in four (1.8%) patients, and two (0.9%) patients died within 30 days of the procedure.

Conclusion

TURP under spinal anesthesia appears to be a safe and effective procedure for high-risk cardiac patients when managed with careful preoperative assessment and intraoperative monitoring. Spinal anesthesia provides a favorable balance between surgical efficacy and cardiovascular safety in this vulnerable population. Further multicenter prospective studies are recommended to confirm these findings and develop standardized perioperative care protocols.

## Introduction

Benign prostatic hyperplasia (BPH) represents one of the most common urological conditions affecting elderly men globally, with its prevalence notably increasing after the age of 60 years [1]. With further prolongation of life expectancy, more and more older men are diagnosed with symptomatic BPH that needs surgical treatment. Transurethral resection of prostate (TURP) has been the standard procedure for moderate to severe BPH as it is effective in relieving obstruction of urine and enhancing the life quality [2]. Nevertheless, medical comorbidities, especially cardiovascular diseases, are quite high in patients undergoing TURP, and this presents a challenge to the perioperative planning and management [3]. The most common type of perioperative morbidity and mortality in non-cardiac surgery is cardiovascular disease, and individuals at risk of ischemic heart disease, congestive heart failure, valvular heart disease, or arrhythmia are a special concern or challenge during urological surgery procedure like TURP [4]. The high-risk cardiac patients include patients of American Society of Anesthesiologists (ASA) physical status III or IV with or without a recent myocardial infarction or poorly controlled heart failure or unstable angina. This risk predisposes to the occurrence of cardiac events during surgery (perioperative cardiac events), with myocardial infarction, rhythm arrhythmias, heart failure events, and even sudden cardiac death being among the most common problems initiated by these conditions [5]. Anesthetic technique is significant in determining the outcome of patients. Although general anesthesia was traditionally used, spinal anesthesia is now considered preferable when undertaking TURP surgeries, improved by its benefits including persistent respirations, decreased thromboembolic hazard and successful analgesia with a minimum influence of the surgical stimulation induced craving reaction [6]. Some theoretical advantages of spinal anesthesia in high-risk cardiac patients are restricting sympathetic stimulation, maintaining myocardial oxygen supply-demand ratio, and preventing the cardiodepressive effect that is produced by the majority of general anesthetic agents [7]. However, the spinal anesthesia has its share of worries [8]. Blockade of sympathetic outflow through the spinal anesthesia may cause severe hypotension and bradycardia, both of which are especially unhealthy in patients with compromised cardiac reserve [9]. In this subset, close titration of the dose of anesthetics, preloading techniques, and intraoperative hemodynamic monitoring should be done vigilantly [10]. Also, TURP itself comes with risks, as there is the possibility of TUR syndrome, which is a life-threatening condition caused by excessive absorption of irrigation fluids, resulting in hyponatremia, fluid overload, and neurological manifestations. The treatment approaches for these risk factors among the cardiac-compromised people are multidisciplinary and involve urologists, anesthesiologists, and cardiologists [11].

Objective

To assess intraoperative hemodynamic changes (including hypotension, bradycardia, and vasopressor use) and postoperative cardiac events (such as arrhythmias, heart failure, and myocardial infarction) within 30 days following TURP under spinal anesthesia in high-risk cardiac patients, with the aim of evaluating the safety and feasibility of this anesthetic approach in a vulnerable surgical population.

## Materials and methods

This was a descriptive observational study conducted at the Department of Urology, Fatima Jinnah Medical University (FJMU)/Sir Ganga Ram Hospital, Lahore, from February 2023 to August 2023. A total of 225 male patients aged 50 years and above were included in the study. The sample size was determined using the WHO sample size calculator, with assumptions based on an anticipated complication rate of 15% in high-risk cardiac patients undergoing TURP, a 5% margin of error, and a 95% confidence level. Data were collected through a non-probability consecutive sampling technique. The study included male patients aged 50 years and above who were diagnosed with benign prostatic hyperplasia requiring surgical intervention through TURP. Only those with a documented high-risk cardiac status were considered eligible, encompassing conditions such as ischemic heart disease, congestive heart failure, valvular heart disease, arrhythmias, or those with a history of coronary interventions, including stenting or bypass surgery. All patients were classified as American Society of Anesthesiologists (ASA) physical status class III or IV. Patients were excluded if they were deemed unfit for spinal anesthesia due to contraindications such as coagulopathy or significant spinal deformities. Additional exclusion criteria included the presence of neurological conditions affecting lower limb sensation or motor function, active urinary tract infection at the time of surgery, or if the TURP procedure was performed under general anesthesia or using a combined spinal-epidural technique.

Data collection procedure

All patients underwent a comprehensive preoperative assessment that included detailed medical history, physical examination, routine laboratory investigations, electrocardiography (ECG), and echocardiography. 

A consultation with a cardiologist was made official to state the cardiac risk and before the surgery, the medical condition of the patient should be streamlined. This assessment aimed at determining whether there were any risks that could be altered and that the patient was going to be safe. All the patients received spinal anesthesia with 0.5% hyperbaric bupivacaine and the allowance of dosage was carefully adjusted to the respective weight of patients and their clinical status. The desired depth of the sensory block was as far as T10 dermatome. During the process, non-invasive blood pressure measurement, ECG monitoring, and pulse oximetry were used to monitor the patient in case of early identification of hemodynamic instability. During surgery, intraoperative documentation was done; time of surgery was recorded in minutes, blood loss approximated in milliliters, and the presence of intraoperative hypotension indicated as blood pressure below 90 mmHg. The heart rate that was less than 50 beats per minute was recorded as bradycardia. Vasopressors and atropine were also noted, and the rate of volumes of fluid that was used in the procedure was mentioned as well. Post-operative outcomes that were recorded were length of stay hospital in days, development of new cardiovascular events like cardiac arrhythmia, heart failure or acute myocardial infarction, occurrence of TUR syndrome, reoperation between 0 and 30 days, and 30 days of post-operative mortality.

Data analysis

Data were entered and analyzed using SPSS software version 26.0 (IBM Corp., Armonk, NY). Continuous variables such as age, surgery duration, and blood loss were presented as means with standard deviations. Categorical variables such as incidence of hypotension or cardiac events were reported as frequencies and percentages. A p-value of less than 0.05 was considered statistically significant throughout the analysis.

## Results

The mean age of the participants was 68.4±7.8 years. A majority, 159 (70.7%), were classified as ASA Class III, while 66 (29.3%) belonged to ASA Class IV, indicating a high-risk surgical cohort. Among cardiac comorbidities, ischemic heart disease was present in 142 (63.1%) participants, congestive heart failure in 51 (22.7%), arrhythmias in 19 (8.4%), and valvular heart disease in 13 (5.8%). Regarding the burden of cardiac conditions, 108 (48.0%) had a single cardiac condition, 77 (34.2%) had two, and 40 (17.8%) had three or more (Table 1).

**Table 1:** Demographic and Baseline Characteristics of Study Participants (n=225) ASA: American Society of Anesthesiologists; ACE: angiotensin-converting enzyme; ARB: angiotensin receptor blocker; SD: standard deviation

Characteristic	Value	Percentage (%)
Age (years), Mean±SD	68.4±7.8	–
ASA Class III	159	70.7%
ASA Class IV	66	29.3%
Ischemic Heart Disease	142	63.1%
Congestive Heart Failure	51	22.7%
Arrhythmias	19	8.4%
Valvular Heart Disease	13	5.8%
Cardiac Comorbidity		
Single Cardiac Condition Only	108	48%
Two Cardiac Conditions Combined	77	34.2%
Three or More Cardiac Conditions	40	17.8%
Total	225	100%
Medication profile (Pre-surgery)		
Beta-Blockers	137	60.9%
ACE Inhibitors/ARBs	121	53.7%
Antiplatelet Agents (e.g., Aspirin)	144	64%
Statins	112	49.7%
Diuretics	88	39.1%

The mean surgery duration was 58.6±12.3 minutes and the mean estimated blood loss was 180±75 mL. Intraoperative hypotension occurred in 63 (28%) of the cases, while bradycardia was noted in 27 (12%) of patients. Vasopressor support was required in 52 (23.1%) of the surgeries. The mean total crystalloid infused was 1420±350 mL, and 19 (8.4%) participants required a blood transfusion, with an average of 1.4±0.6 units transfused (Table 2).

**Table 2:** Intraoperative Outcomes SD: Standard deviation.

Intraoperative Variable	Value
Surgery Duration (minutes), Mean±SD	58.6±12.3
Estimated Blood Loss (mL), Mean±SD	180±75
Intraoperative Hypotension	63 (28%)
Bradycardia	27 (12%)
Vasopressor Support Required	52 (23.1%)
Total Crystalloid Infused (mL)	1420±350
Blood Transfusion Required	19 (8.4%)
Number of Units Transfused (if any)	1.4±0.6 units

The mean hospital stay was 3.7±1.6 days. TUR syndrome occurred in five (2.2%) patients and four (1.8%) required a reoperation within 30 days. New-onset arrhythmias were seen in 11 (4.9%) and acute heart failure in seven (3.1%) patients. Non-fatal myocardial infarction occurred in three (1.3%) participants, while 30-day mortality was observed in two cases (0.9%) (Table 3).

**Table 3:** Postoperative Outcomes TUR: Transurethral resection; SD: standard deviation.

Postoperative Variable	Value	Percentage (%)
Hospital Stay (days), Mean±SD	3.7±1.6	–
TUR Syndrome	5	2.2%
Reoperation within 30 Days	4	1.8%
New-Onset Arrhythmias	11	4.9%
Acute Heart Failure	7	3.1%
Non-Fatal Myocardial Infarction	3	1.3%
30-Day Mortality	2	0.9%

The chi-square analysis demonstrated a statistically significant association between the type of cardiac comorbidity burden and postoperative complications (χ²=16.27, df=2, p=0.0002). Patients with multiple comorbidities, particularly those with ischemic heart disease (IHD), congestive heart failure (CHF), and arrhythmia or valvular disease, had a much higher complication rate (40%) compared to those with IHD alone (20%) (Table 4).

**Table 4:** Association Between Type of Cardiac Comorbidity Burden and Postoperative Complications IHD=Ischemic heart disease; CHF=congestive heart failure Statistically significant at p<0.05.

Comorbidity Burden	Complications Present (n = 30)	No Complications (n = 195)	Chi-square (χ²)	df	p-value
IHD Only	6 (20.0%)	102 (52.3%)	16.27	2	0.0002
IHD+CHF	12 (40.0%)	65 (33.3%)			
IHD+CHF+Arrhythmia/Valve Disease	12 (40.0%)	28 (14.4%)			
Total	30 (100%)	195 (100%)			

## Discussion

This study evaluated the outcomes of TURP under spinal anesthesia in a cohort of 225 high-risk cardiac patients, focusing on both intraoperative safety and postoperative recovery profiles. Our results indicate that TURP done under spinal anesthesia is a relatively safe and possible procedure with reasonable cardiovascular, surgical complication rates in this clinical group. The age of the study members was 68.4 years, which agrees with the epidemiological trends that BPH cannot be surgically treated usually among older men, with a large number of comorbidities such as cardiac disease [12]. The most prevalent comorbidity was IHD in our cohort, and this observation is in line with literature findings, which had highlighted coronary artery disease as the highest cardiac risk factor in urology patients due to old age [13].

During surgery, 28% of the patients had hypotension, and 12% developed bradycardia, which were anticipated responses of the patient's body to spinal anesthesia, especially considering the poor cardiac condition at that time [14]. But they were handled adequately by vasopressors and atropine in all the instances, and there was no necessity of conversion to general anaesthesia. This will reinforce the hypothesis that spinal anesthesia could be endured even by ASA class III-IV patients, but with careful anesthesia and observation [15]. We had a marginally higher incidence of hypotension and bradycardia compared with low-risk cardiac patients who underwent TURP with spinal anesthesia, further reiterating the fact that high-risk cardiac patients are more susceptible in such conditions than the rest of the population, as mentioned in previous studies. The lower complication and mortality rates in this study may be attributed to strict preoperative cardiac optimization, the use of spinal anesthesia to reduce cardiovascular stress, and experienced surgical and anesthesia teams at a high-volume tertiary center [16]. There were manageable limits of postoperative complications. In our study, 4.9% of patients developed new arrhythmias and 1.3% experienced cardiac events, including non-fatal myocardial infarction. In comparison, the international literature reports cardiac event rates of around 57% after non-cardiac surgery in similar high-risk patient cohorts [17]. Remarkably, the rate of TUR syndrome was 2.2%, which is not inferior to the rate accepted worldwide to be 12 in the modern practice of TURP and implies that the typical TUR procedure in our hospital is in line with the safety standards established across the world [18]. The reoperation rate within 30 days (1.8%) and the 30-day mortality rate (0.9%) were low, especially considering the high-risk nature of the study population. Previous research on TURP in cardiac-compromised patients reported 30-day mortality rates ranging from 1% to 3%, depending on the extent of pre-existing cardiovascular disease and perioperative management protocols. Our outcomes suggest that with proper preoperative cardiac assessment, vigilant intraoperative monitoring, and structured postoperative care, TURP can be performed with an acceptable risk profile even in patients previously considered borderline surgical candidates. Furthermore, our study highlighted that a significant proportion of patients were on medications such as beta-blockers, angiotensin-converting enzyme (ACE) inhibitors, and antiplatelet agents, necessitating careful perioperative medication management. Temporary discontinuation of antiplatelets, balancing thrombotic versus bleeding risks, was performed as per cardiology consultation, underscoring the importance of multidisciplinary collaboration. 

Limitations

This study has several limitations that warrant consideration. First, its single-center design may limit generalizability, as surgical techniques, anesthetic practices, and postoperative care protocols can vary across institutions. Second, the observational nature of the study introduces potential for selection bias, particularly as patients who were extremely frail or deemed unfit for spinal anesthesia may have been excluded, thereby skewing outcomes toward a more stable subset of high-risk individuals. Third, no control group of low-risk cardiac or general BPH patients was included for direct comparison, limiting our ability to quantify the relative risk. Additionally, we did not assess long-term outcomes such as delayed cardiac events or TURP-related complications beyond 30 days.

## Conclusions

It is concluded that TURP under spinal anesthesia is a safe and effective surgical option for patients with high-risk cardiac profiles, provided that careful patient selection, preoperative optimization, and vigilant intraoperative monitoring are ensured. Despite the increased vulnerability to hypotension, bradycardia, and perioperative cardiac events in this group, the overall rates of major complications, reoperations, and 30-day mortality remained low and within acceptable clinical limits. Our study demonstrates that spinal anesthesia can be favorably utilized in high-risk cardiac patients undergoing TURP, balancing the need for effective surgical intervention to minimize cardiovascular stress.
